# Synthetic biology approaches to biological containment: pre-emptively tackling potential risks

**DOI:** 10.1042/EBC20160013

**Published:** 2016-11-30

**Authors:** Leticia Torres, Antje Krüger, Eszter Csibra, Edoardo Gianni, Vitor B. Pinheiro

**Affiliations:** ^1^ Department of Structural and Molecular Biology, University College London, Gower Street, London, WC1E 6BT, U.K.; ^2^ Birkbeck, Department of Biological Sciences, University of London, Malet Street, WC1E 7HX, U.K.

## Abstract

Biocontainment comprises any strategy applied to ensure that harmful organisms are confined to controlled laboratory conditions and not allowed to escape into the environment. Genetically engineered microorganisms (GEMs), regardless of the nature of the modification and how it was established, have potential human or ecological impact if accidentally leaked or voluntarily released into a natural setting. Although all evidence to date is that GEMs are unable to compete in the environment, the power of synthetic biology to rewrite life requires a pre-emptive strategy to tackle possible unknown risks. Physical containment barriers have proven effective but a number of strategies have been developed to further strengthen biocontainment. Research on complex genetic circuits, lethal genes, alternative nucleic acids, genome recoding and synthetic auxotrophies aim to design more effective routes towards biocontainment. Here, we describe recent advances in synthetic biology that contribute to the ongoing efforts to develop new and improved genetic, semantic, metabolic and mechanistic plans for the containment of GEMs.

## Introduction–what's the risk?

We have altered our environment throughout history. We have domesticated animals and plants, we have invented ever more sophisticated tools, and we have harnessed nearly every possible resource available on Earth to ensure our continued survival and fuel our evolving needs and lifestyles–not always ethically, not always sustainably, rarely understanding or minimizing the impact of our actions.

We are now at a point where our knowledge and technology enable us to change biology into a tool itself–the first tool that can exceed its own design parameters as life can repair itself, multiply, evolve and potentially escape. A tool escaping implies the potential entry of engineered organisms (or engineered genetic information) into the environment, and a possible detrimental impact on the environment, whether direct (e.g. competing with natural species) or indirect (e.g. changing the balance between native species). The potential environmental effect of genetically modified organisms (GMOs) has been a long-standing concern for scientists and a regular topic of discussion since the dawn of molecular biology in the mid-1970s [1,2].

The consensus approach is containment–any strategy that can be deployed to prevent GMOs (or their genetic information) from escaping the controlled experimental environment. Containment is underpinned by our understanding of risk and by the tools available to implement it.

In its most basic definition, risk is the probability of a known possible event. Complex situations, where probabilities may not be determined or where we cannot foresee every possible event, undermine the reliability of risk estimation [3]. It affects our judgement of a situation that becomes increasingly subjective and may account for some of the disparity in acceptance of GMOs seen across societies.

Lack of specific knowledge is not an impediment if pre-emptive action can be taken to address the sources of potential risk. For instance, although we may not be able to foresee how every GMO can interact with the environment and how likely those scenarios are, we can still tackle its potential sources of risk to the environment, i.e. GMOs interacting with the environment as organisms or the information coded by the GMO (e.g. antibiotic resistance [4]) being passed onto the environment.

Containment naturally emerges from that framework and its goals are clear: avoid, prevent and minimize–avoid known traits that are likely to benefit GMOs in the natural environment, prevent GMOs from entering the environment and minimize any potential penetration into the environment. The same criteria are also applicable to any genetic information used in engineering an organism since exchange and acquisition of genetic information, better known as horizontal gene transfer, particularly for microorganisms, is common in the environment [4,5].

This review focuses on the biological containment of genetically engineered microorganisms (GEMs) and, in particular, on recent synthetic biology developments that have the potential to greatly enhance containment standards. This being a short review, we apologize to colleagues whose work we did not include and our aim is to complement a number of excellent recent reviews on biological containment [2,5–9].

## Established containment strategies

A number of strategies have been implemented over the years to establish biological containment, which is described as successful if the probability of an organism bypassing the containment measures drops below 10^−8^ [1,10,11]–that is, recovering fewer than 10^2^ CFU from a 100 mL culture at O.D._600_ of 1 (given a microorganism for which an O.D._600_ of 1 equates to 10^8^ CFU/mL).

**Figure 1:**
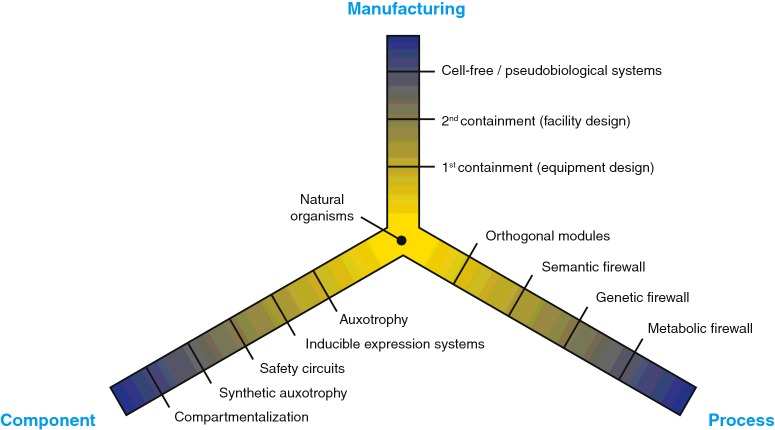
Routes to biological containment Strategies to prevent the escape of GEMs can be broadly divided between changes to the manufacturing of biological products, changes to individual components or changes to biological processes themselves. Within each approach, different strategies are ranked by the expected engineering challenge to implement them in bacteria (yellow-to-blue scale for increasing engineering challenge). Compartmentalization [21–23] and cell-free systems [24,25] are not covered in this review.

The current gold standard in containment, as it is implemented for the industrial-scale production of microorganisms (and microorganism-derived products), is achieved by the design of physical barriers. Engineering design of equipment, process and production plant all contribute to the containment of GEMs [12], as shown in Figure 1.

In addition to physical measures, containment can be achieved through biological means. The three most established strategies are based on inducible systems, auxotrophy and cellular circuits [13]. Crucially, no single strategy used in isolation has delivered, to date, successful containment [5].

Inducible systems, the least effective route towards containment, include well-characterized gene expression platforms widely used in molecular biology (e.g. tetracycline promoter regulation with anhydrotetracycline [14]). Containment is achieved by the requirement of an inducer of gene expression not common in the environment. Thus, the engineered traits are displayed only in the controlled growth environment of the lab when the inducer is present. Outside that environment, the engineered trait is not displayed and cells lose any potential advantage granted by the inducible system.

Auxotrophy removes the ability of the organism to synthesize a vital compound that must be acquired from the growth media or the environment. Most bacterial laboratory strains have some form of auxotrophy, which reduces their fitness and contributes to containment in the laboratory setting. Targeting compounds central for cell survival and that are required in high concentrations maximize the effectiveness of the approach, e.g. thymine in *thyA*^−^ mutants [15–17]. Notably, inducible systems or auxotrophy do not address the potential risk of genetic information escape, i.e. horizontal gene transfer.

Cellular circuits include kill switches as well as addiction modules, all achieving containment by making cells dependent on a compound (or lack thereof) or particular genetic information. Kill switches are genetic circuits that once activated lead to cell death, such as through the expression of the membrane-disruptive proteins of the Hok family [18].

These circuits can be used in a number of different topologies enabling complex logic circuits to be built that require particular combinations of inputs for cell survival [19,20].

Addiction modules, or toxin-antitoxin systems, in contrast, consist of a combination of stable toxin and an unstable antitoxin (reviewed by Hayes [26]) with cell survival balanced between the production and degradation of both. For containment, toxin and antitoxin genes can be placed in different parts of the cell's genetic repository, e.g. with the toxin on the plasmid and antitoxin on the genome. Such arrangement ensures that in case of horizontal gene transfer, the plasmid carries the toxin but not the antitoxin, killing the new host and limiting the penetration of any genetic information stored in the plasmid.

## Complex biological containment circuits

Used in isolation or reliant on a single target, the more established containment strategies are not sufficient to prevent escape because of the low evolutionary cost of bypassing or reverting the containment mechanism. For instance, most containment circuits developed by Chan and colleagues consisting of a single toxin [20], did not achieve escape frequencies of less than 10^−6^, with mutation of the lethal actuator being one of the main sources of escape.

The evolutionary cost of escape can be increased, and with it the efficacy of containment, by combining multiple targets or containment strategies–each individual measure described as a layer of containment. Chan and colleagues did this by introducing two different lethal actuators and pushing escape frequencies below 10^−8^ even after 4 days [20]. Crucially, although layers of containment are additive, they do not show synergism.

In another example of multi-layered containment, Wright and colleagues developed a complex circuit (GeneGuard) that relies on conditional origins of replication, auxotrophy and an addiction module for containment [27]. The GeneGuard circuit is split between a genomic and a plasmid module, ensuring inter-dependence of plasmid and host (the plasmid requires a genomic factor for replication, the host requires auxotrophic supplementation from the plasmid) and minimizing information escape (the plasmid harbours toxin of the addiction module). The work demonstrates that each factor contributes to containment but it is less clear whether together they would reach a deemed safe level of containment (see above).

Gallagher and colleagues also pursued a multifactor approach to containment, integrating riboregulators (requiring multiple inputs to allow translation of essential genes), engineered addiction modules (based on nuclease and methylase systems), auxotrophy and supplemental repressors (to further limit system mis-activation by increasing its silencing) [28]. A strain engineered with a four-layer containment demonstrated robust long-term containment with escape frequencies of less than 2 x 10^−12^–the lowest reported to date.

## Engineering further containment: trophic containment

Biology is not limited by what is natural, but rather, by what is possible. As such, it is feasible to engineer novel approaches for containment that exploit alternative core biological processes, including metabolism, translation and genetic information storage.

Metabolic containment, in which the organism depends on the supply of a metabolite for survival, is exemplified by auxotrophism (detailed above). A key shortcoming of auxotrophism however, is that metabolites are generally common between many organisms and can thus be found in the natural environment, together with the genes for their biosynthesis. Nevertheless, this approach can be extended by creating organisms that require synthetic compounds for survival. Because these synthetic cofactors are not available in nature, containment of such a synthetic auxotroph should outperform the usual auxotrophic barriers.

Non-canonical amino acids (ncAAs) have recently been reported as viable synthetic cofactors by two groups. Lajoie and colleagues built a synthetic *Escherichia coli* auxotroph by engineering six essential genes (*adk*, *alaS*, *holB*, *metG*, *pgk* and *tyrS*) to depend on the ncAA L-4,4’-biphenylalanine (BFA). Computer-assisted protein engineering was used to identify sites in essential proteins where hydrophobic cores could be engineered to require BFA, which differs significantly in size and geometry from the canonical set of amino acids. Starting from an *E. coli* strain devoid of amber stop codons [29], BFA was added to the genetic code of the host through an engineered transfer ribonucleic acid (tRNA) and aminoacyl-tRNA synthetase pair targeted to amber codons [30]. As with other strategies, a single containment layer did not provide escape frequencies below 10^−8^, especially in extended culture conditions (7 days). Once multiple dependencies were created however, escape frequencies rapidly dropped below 2 x 10^−12^, their limit of detection, even after 14 days.

Using an analogous approach (i.e. using engineered genomic amber sites to target ncAA incorporation into essential genes), Rovner and colleagues explored a small number of strategies to identify suitable target sites for ncAA incorporation. In addition to computer-assisted design, they also targeted residues proximal to the N-terminus and in the active site of essential host factors [31]. The result was also the isolation of strains that in the presence of multiple containment layers had escape frequencies below 10^−8^ even after prolonged culture.

However, ncAAs are not the only viable synthetic cofactors. Allosteric regulation of enzymes through small molecules is widely present in biology [32], can be exploited with synthetic compounds [33] and can also be engineered [34,35]. Building on the work of Deckert and colleagues [35], Lopez and Anderson established a directed evolution platform (SLiDE–synthetic auxotrophs based on ligand-dependent essential genes) for the selection of engineered *E. coli* essential proteins that require external complementation from small molecules [36].

Using SLiDE, Lopez and Anderson engineered an *E. coli* BL21 strain with three essential genes (*tyrS, metG, pheS*) dependent on benzothiazole for function. The approach is scalable and as the authors point out, considerably cheaper than whole genome recoding. The escape frequency of the triple mutant was below detection (less than 3 x 10^−11^) but rose to around 10^−6^ after a couple of days. Still, SLiDE could be used to target more than three genes or to rely on chemical compounds that are structurally or chemically more distant from biological metabolites. Interestingly, there are now reports that sites for allosteric regulation may be structurally conserved between homologous proteins [37], allowing developments made in model organisms to be rapidly adapted to other industrially relevant species.

In theory, containment by auxotrophy can be extended beyond chemical supplementation, with hosts being engineered not only to rely on a synthetic cofactor but also to have a synthetic metabolism, which could conceivably create a barrier to information transfer between organism and biology. If the engineered metabolism differs sufficiently from the natural system, they could become incompatible leading to the accumulation of undesired intermediates or even toxic compounds in a natural host.

Some proteins naturally require cofactors, such as specific metal ions, vitamins and redox centres. In principle, it should also be possible to engineer these proteins to depend on alternative cofactors, developing a new class of synthetic auxotrophs. Metalloprotein engineering has come closest to this approach, creating the environment for non-biological chemistries and raising the possibility of introducing those *in vivo* [38–40].

Nevertheless, containment would require those reactions to be linked to the organism's core metabolism to cement the auxotrophic requirement; an idea explored by the UCL iGEM 2014 team with synthetic quinones [41]. Quinones have a central role in the transport of electrons across membranes and therefore in the energy metabolism of the cell. The team proposed that host factors could be engineered to depend on synthetic quinone-like compounds; an idea that has not yet been explored further.

## Engineering further containment: semantic containment

While genetic circuits and trophic containment can be effective strategies to stop GEMs from proliferating into the environment, they do less to prevent the escape of the engineered genetic information. For that, the information itself has to be altered–making the genetic code and genetic information storage important targets for containment.

**Figure 2:**
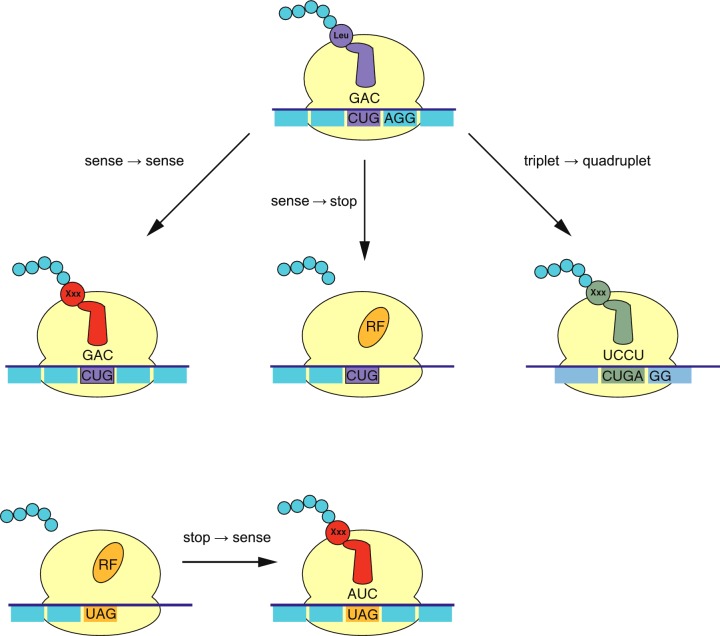
Changing the genetic code for semantic containment Any change in the genetic code–sense-to-sense, sense-to-stop or triplet-to-quadruplet (see orthogonal translation as a containment strategy section)–leads to the incorporation of a different amino acid (Xxx) or protein synthesis termination (RF–release factor). As a result, the same messenger RNA (mRNA) message leads to two different proteins under the natural and engineered code. If the reassignment is sufficiently disruptive, such that under the natural code the resulting protein is not functional, then information cannot move from engineered to natural organism.

The genetic code is universal (barring a few minor exceptions) hence, in principle, genetic information can be translated into the same protein regardless of the host organism. Containment emerges if an engineered organism can operate under a different genetic code, either through codon reassignment or by altering the decoding rules–see Figure 2. Such an altered code changes the meaning of the genetic information, ensuring functional proteins could only be functional in the engineered host–making the information itself semantically contained.

Codon assignment is primarily established by the specificity of aminoacyl-tRNA synthetases (aaRS) and their cognate tRNAs but further enforced by a number of cellular factors such as additional proofreading proteins [42–47], elongation factors and the ribosome itself [48]. Codon reassignments can take three routes–sense-to-stop, stop-to-sense and sense-to-sense replacements–with modifications of aaRS/tRNA pairs an efficient route towards creating any alternative code [49].

Stop-to-sense codon reassignment, also termed stop codon suppression, occurs naturally in organisms harbouring tRNAs that have evolved to recognize stop codons. When transcribed, those tRNAs–known as suppressor tRNAs–compete with release factors binding the ribosome, delivering an amino acid that is incorporated into the nascent polypeptide and allowing translation to continue [50]. Stop codon suppression has been very successfully exploited to expand the genetic code, enabling the incorporation of a large number of ncAAs through the engineering of aaRS and cognate tRNA that can specifically charge the desired ncAA but that do not interfere with the cellular aaRS or tRNAs (i.e. an orthogonal aaRS/tRNA pair) [45–47].

Still, the competition between stop codon suppression and termination is usually biased towards the highly efficient canonical processes, resulting in poor ncAA incorporation and creating a bottleneck for the large-scale synthesis of ncAA-containing engineered proteins [45–47]. Deletion of release factor 1 (RF1) in *E. coli*, which is possible because of partial redundancy with RF2, significantly improves stop codon suppression and increases ncAA-containing protein yields [51,52].

Stop codon reassignment within an essential gene (for the incorporation of natural amino acids) is, in principle, enough to provide containment, since in the absence of a charged suppressor tRNA, the engineered information cannot be effectively translated and the host dies. Nevertheless, containment can be further improved by placing the stop codon at a site relevant for protein function where the amino acid being introduced cannot be easily substituted. Using ncAAs not abundant or not present in nature can add a further layer of containment, since in addition to the semantic barrier, an auxotrophic barrier is created, with very promising results [31,53]–see above.

Sense-to-sense reassignment can also be used to establish biological containment, but this strategy has not yet been as widely explored. Such reassignment does not have to be direct and can be implemented via sense → stop → sense reassignments–with the recent removal of multiple codons from *E. coli* [54] an intermediate in that process. Any change to the genetic code affects the host's fitness, thus, the engineering challenge increases with the frequency of codon exchanges across a genome. Amber stop codons (TAG), described above for stop codon suppression, occur only 321 times in the *E. coli* genome, and its suppression or reassignment causes minimal impact on cell fitness [52]. Sense codons are naturally more frequent (e.g. 4228 instances of AGG/AGA in the *E. coli* genome; [55]). Hence, unless the synthesis of tailored proteins cannot be engineered to work in parallel with the main cellular processes (e.g. either through compartmentalization, like expression in microalgae chloroplasts [56], or by introduction of a completely orthogonal translation system), reassigning sense codons will lead to an experimental intermediate where the code has at least one degeneracy; meaning more than one aaRS/tRNA pair targeting the incorporation of different amino acids at the same codon. Degeneracy of the genetic code can be advantageous in some circumstances [57], but would more probably lead to protein misfolding and thereby increased burden on the host–the underlying principle of semantic containment.

This presents two alternative approaches to pursue sense-to-sense reassignment: engineer the host to pre-emptively remove degeneracies, or exploit acceptable or transient degeneracies to develop the code. Lajoie and colleagues have demonstrated that rare codons in *E. coli* can be removed from essential genes [52] and have more recently demonstrated that it is possible to scale that process up to large sections of the genome [54], freeing up seven codons that can be reintroduced as novel amino acids or stop codons.

Some degeneracy can be tolerated by the cell sufficiently for it to be of use in the engineering of the genetic code, particularly if the recoding is required for cell survival. For instance, Döring and Marlière probed if cysteine could invade isoleucine and methionine codons by introducing tRNACys variants modified in their anticodon sequences [58]. They coupled the resulting genetic code degeneracy to a mutant ThyA (thymidylate synthetase), with its catalytic cysteine miscoded in the gene. The result demonstrated that all four anticodon variants could support functional ThyA synthesis, and therefore cell survival in growth media lacking thymine, when the anticodon and engineered codon matched. Interestingly, on removal of the selective pressure, the degeneracy persisted for over 500 generations before dropping to below 1% of the population–highlighting that the degeneracy (e.g. AUC → I/C) generated only minimal toxicity.

Other acceptable degeneracies have been reported, such as phenylalanine and 3-(2-naphthyl)-L-alanine [59], or arginine (rare codon AGG) and L-homoarginine [60]. Both teams introduced orthogonal aaRS/tRNA pairs with improved anticodon-codon affinities in *E. coli* (Watson–Crick pairing rather than wobble in the codon-anticodon), thereby outcompeting the natural codon assignment [59]–which can be further fine-tuned by altering host tRNA levels. Mukai and colleagues further minimized the impact of the recoding of arginine to L-homoarginine by making the reassignment temperature dependent and by recoding 38 AGG to other arginine codons in 32 essential genes [60]. More frequent codons can also be reassigned using a similar strategy. Ho and colleagues invaded both serine and leucine codons with an engineered pyrrolysyl-tRNA synthetase capable of charging the ncAA 3-iodo-L-phenylalanine to its cognate tRNA [61].

In an alternative strategy, Hoesl and colleagues used serial passaging to systematically replace tryptophan with L-beta-(thieno[3,2-b]pyrrolyl)alanine (Tpa) in the *E. coli* genome [62]. Working on a tryptophan auxotrophic *E. coli* strain, selection was carried out by lowering indole (tryptophan precursor) while maintaining high beta-thieno[3,2-b]pyrrole concentrations. After 264 passages *E. coli* strains were isolated that could use Tpa or tryptophan in their proteomes, thus engineering a viable degeneracy. While it would not be possible to exploit this degeneracy for containment by genetic code expansion, it could be used as a wedge, progressively altering the organism's large aromatic amino acid requirement to the point that tryptophan could no longer rescue the strain.

Semantic containment when enforced through the use of ncAAs, actually impose a double containment: the information is trapped in the altered genetic code and the host is trapped by a *de facto* auxotrophy for the ncAA. The contribution of each measure towards containment can be deconvoluted by also engineering pathways for the *in vivo* synthesis of the ncAA being used. For instance, Mehl and colleagues [63] introduced a *Streptomyces venezuelae* metabolic pathway capable of synthesizing p-aminophenylalanine (pAF) into an *E. coli* strain capable of incorporating pAF in response to an amber stop codon using an engineered orthogonal TyrRS/tRNA pair. The result was a prototroph with an expanded genetic code–one where the impact of semantic containment can be studied separately from the auxotrophic one.

A similar situation (albeit on a different order of magnitude in terms of complexity) could be achieved by inverting the chirality of biology. Although current biology is based on L-amino acids and D-sugars, life would be possible (and equally efficient) in the opposite combination (D-amino acids and L-sugars)–a ‘mirror’ life [64,65].

## Engineering further containment: genetic containment

Life on Earth is underpinned by nucleic acids. The two natural backbones, deoxyribonucleic acid (DNA) and ribonucleic acid (RNA), are biopolymers made from a limited set of possible nucleotide precursors; variants of a common chemical structure: a phosphate group that is linked to a nitrogenous base via a 5-carbon sugar linker. The fact that modern, naturally occurring genetic systems are all based on DNA and RNA does not mean that other chemical forms of genetic information storage are not conceivable. With the aim of finding novel building blocks for the design of artificial nucleic acids, each of the three components of natural nucleotides has been iteratively replaced by alternative chemical structures rendering what has been defined as ‘xeno–nucleotides’ [66]. Modifications to the canonical structure of nucleotides have resulted in numerous variants presenting: unnatural nucleobases, alternative base-pairings, novel cyclic or even acyclic sugar backbones, and altered phosphate groups or alternative leaving groups. In depth reviews regarding the different chemistries developed so far can be found in [67–69].

A subset of the available chemistries has even been successfully polymerized into Xenobiotic Nucleic Acids (XNAs). Some of these XNAs allowed duplex formation [67] and information storage [70,71], and were even evolved into functional molecules such as aptamers and XNAzymes [70–73]. These results suggest that DNA and RNA are not, in principle, the only genetic polymers capable of sustaining life, and so life based on XNAs may also be thinkable.

To date, no life on Earth has been reported to use XNA for genetic information storage and it is unclear whether biology would be able to access the information within it. If not, then the biocontainment of a GEM carrying XNA as genetic material could be feasible. Prevention of horizontal gene transfer to nature could arise from the genetic isolation of XNA-based GEMs. As replication, transcription or incorporation of an XNA into the genome of GEM's natural counterparts would be prevented, a ‘genetic firewall’ could be raised between nature and genetic engineering.

**Figure 3:**
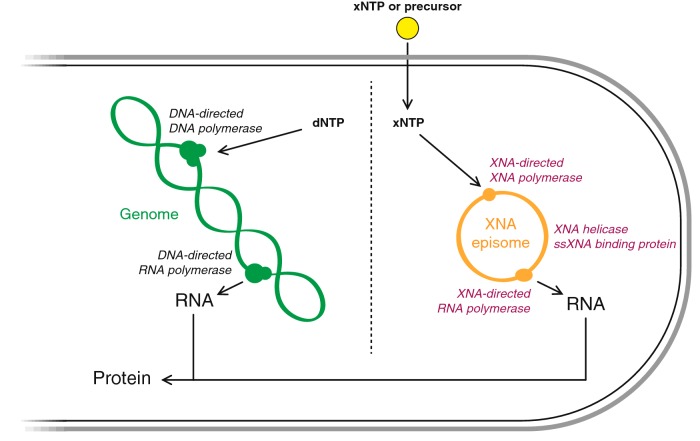
An orthogonal replication system for genetic containment An XNA genetic element (in orange) is maintained by the external provision of xenobiotic nucleoside triphosphates (xNTPs) or cell-permeable precursors (yellow) and replicated by means of an engineered XNA-dependent XNA polymerase (or XNA replicase) and accessory proteins (in purple). Selection of the synthetic episome across generations occurs by encoding a vital gene product or functional XNA in the episome itself. In either case, an XNA-directed RNA polymerase is necessary (in purple). Prevention of cross-talk with the natural DNA genetic system (in green) is essential to create a stable XNA system and to establish an effective genetic firewall (dotted line).

Before even envisaging a GEM built upon XNA, a simpler first approach to develop and test a genetic firewall would be to create an XNA-based replication unit (e.g. episome) that can be maintained in a bacterial cell independently from its DNA genome (Figure 3). Any desired trait could be engineered into the XNA episome while the DNA genome would only be used for chassis maintenance. In order to set up an XNA-episome replication system *in vivo* there are several characteristics that the XNA, the episome itself and the host cell must fulfil (adapted from [74]):

● Regarding the potential **XNA**,

1) to ensure cell survival, the XNA cannot be toxic in any way, be it as precursor, triphosphate, polymer or degradation products.
2) to allow an initial transference of information from natural nucleic acids and *vice versa*, a dedicated XNA synthetase and XNA reverse-transcriptase should be available.
3) to attain orthogonality (complete isolation from the DNA/RNA genetic system), the XNA should not be interpreted by the host cell replication or transcription machinery.

● Regarding the **EPISOME**,

4) to guarantee maintenance in controlled culture conditions, one selectable function indispensable for growth must be encoded in the XNA episome (like antibiotic resistance is encoded in commercial plasmids):

4.1) if the selectable marker encodes an mRNA that is destined to be translated into an essential protein (e.g. detoxification of an antibiotic), then a dedicated RNA polymerase is required.
4.1) if the selectable marker is a functional XNA, this molecule would need to perform a metabolic action vital to the host cell (e.g. XNA molecule that binds and inactivates a small toxic RNA). Such functional XNA must be developed and crucially, any function must be restricted to XNA–the same sequence in a different chemistry cannot rescue selection.

5) to replicate the XNA episome across generations, a dedicated XNA replicase, XNA helicase and XNA single-strand binding protein should be encoded in the episome.
6) to retain orthogonality, XNA enzymes should not accept natural nucleic acids as substrates.

● Regarding the **HOST CELL (e.g.** ***E. coli*****)**,

7) to prevent replication in non-controlled conditions, XNA precursors cannot be made in the cell. Cells must be auxotrophic for the XNA building blocks.
8) to provide cells with XNA precursors, a natural or engineered mechanism for the uptake should exist.

There is a remaining point, regarding the destiny of xeno-nucleotides *in vivo* that has not been included in the list because it does not represent an essential item to establish an XNA episome, but requires attention. Natural nucleic acids are synthesized using nucleoside triphosphates as monomers. However, only the nucleoside monophosphate ends up being incorporated into the DNA or RNA molecule while the remaining pyrophosphate, necessary for the activation of the monomer, is released as a secondary product. As the pyrophosphate is not included in the final biopolymer it is known as the ‘leaving group’. Pyrophosphate is further hydrolysed to release two molecules of phosphate. In nature, no exception to the usage of pyrophosphate as the leaving group has ever been reported. It has been pointed out that it would be difficult to install xeno-nucleoside triphosphates in a cell environment without interfering with DNA and RNA metabolism, cell energy supply [adenosine triphosphate (ATP) levels] or substrate-level phosphorylation (due to the free available phosphate) [74]. One way to overcome this problem would be to use a different type of leaving group in the precursors for the enzymatic synthesis of the XNA. Different chemistries for alternative leaving groups, based on L-amino acids, phosphono-L-Ala or iminodiacetate, have been suggested and studied [75], however the enzymes capable of efficiently incorporating the nucleoside monophosphates out of the alternative nucleotides have yet to be developed.

## Potential XNA-episome chemistries–sugar-modified nucleic acids

Based on the conditions described in the previous section for an XNA to embody an episome *in vivo*, we describe a group of potential candidate chemistries that have the potential to fulfil conditions described in one to three above.

The sugar moiety of natural nucleotides has been extensively modified. Changes in functional groups, size and isomerization states (including cyclical and linear linkers) have been explored returning the following candidate XNAs: anhydrohexitol nucleic acid (HNA) [76], threose nucleic acid (TNA) [77], glycerol nucleic acid (GNA) [78], cyclohexene nucleic acid (CeNA) [79] and locked nucleic acid (LNA) [80,81].

In order to study the physico-chemical and biological properties of the XNAs, a mechanism of polymerizing xeno-nucleotides is necessary. Polymerization can occur chemically, through solid-phase synthesis, or enzymatically via polymerases. Chemical polymerization of xeno-nucleotides is generally effective enough for assembling short oligomers. On the other hand, natural polymerases have a stringent substrate specificity that prevents the efficient incorporation of chemically modified nucleotides. Thus, in order to study the potential applications of XNAs it is always necessary to develop the enzymatic machinery that can cope with XNA polymerization and XNA interpretation (i.e. information propagation). The way to do this is either by screening natural polymerases for such activity, or by engineering known polymerases through rational design or directed evolution. An example of engineering is the Archaean 9°N DNA polymerase that was originally modified by the introduction of the A485L mutation (commercially known as Therminator, New England BioLabs, Inc. [82]) to incorporate a wide range of unnatural nucleotides. Under optimal conditions and provided that no long poly-G stretches are present, this enzyme is capable of copying DNA templates into TNA with high fidelity [83]. On the other hand and as a result of polymerase screening, TNA retro-transcriptase activity was discovered in the wild-type DNA polymerase I of *Geobacillus stearothermophilus* (Bst) showing about 60% full-length conversion of TNA into the complementary DNA product [84].

In a more extensive work of engineering carried out by directed evolution and the application of a novel *in vitro* selection platform, a different Archaean polymerase from *Thermococcus gorgonarius* (TgoT) was evolved into a synthetase able to transcribe long stretches of DNA into HNA (Pol6G12) [71]. The selection also provided two other enzymes (PolC7 and PolD4K) with broader substrate specificity capable of synthesizing CeNA and LNA among other chemistries [71]. In order to reverse transcribe the XNA polymers back into DNA, TgoT was also engineered by rational design into two retro-transcriptases able to copy HNA and TNA (RT521) or CeNA and LNA (RT521K) back into DNA [71]. This was a step forward in the study of XNAs, not only because several novel enzymes were engineered but also because an *in vitro* platform for the selection of XNA polymerases–adaptable to other XNA modifying enzymes–was devised, opening the possibility of the evolution of more enzymes of interest such as XNA replicases, XNA-directed RNA polymerases, XNA ligases and XNA polinucleotide kinases [85].

At a translational level, the compatibility of the above XNAs with the protein synthesis machinery of *E. coli* has only been tested for HNA [86]. A short messenger RNA was designed, with the first and second codons replaced by HNA. Efficient peptide formation and translocation was observed in translation experiments performed *in vitro*. Ribosomal binding of the messenger and tRNA incorporation showed no differences between a fully RNA molecule and the HNA/RNA hybrid described [86].

## Potential XNA-episome chemistries–modified nucleobases and alternative base-pairings

A different category of xeno-nucleotides is defined by those carrying nucleobase modifications, resulting in the diversification of nucleotide pairing schemes beyond those known to nature and described by Watson and Crick. Natural nucleobases are paired based on the rules of size complementarity (large purine pairs with small pyrimidines) and hydrogen-bonding complementarity (hydrogen-bond donor pairs with hydrogen-bond acceptors). However, if the hydrogen-bond donating and acceptor groups are shuffled, eight novel nucleobases capable of forming four additional base-pairs with the same geometry as A:T and C:G pairs could be envisaged. This was the work pioneered by Yang and colleagues, who described a family of nucleoside analogues, and probed that at least two of them (trivially called dP:dZ) could be a suitable alternative base-pair *in vitro* [87]. The artificial dP (2-aminoimidazo[1,2-a]-1,3,5-triazin-4(8H)-one) and dZ (6-amino-5-nitro-2(1H)-pyridone) nucleotides are able to pair with three hydrogen bonds and standard Watson–Crick geometry. The *Thermus aquaticus* DNA polymerase was engineered to improve the synthesis of a nucleic acid containing both dP and dZ along with the four canonical dNTPs (ATCGPZ_DNA) [88]. When incorporated into DNA the unnatural base-pair is polymerase chain reaction (PCR) amplifiable with a fidelity of 99.8% [89]. The ribonucleotide versions of dP and dZ (rP and rZ, respectively) were also synthesized and efficiently incorporated into RNA using T7 RNA polymerase. To retrieve the information back into an ATCGPZ_DNA molecule, a superscript reverse-transcriptase was used [90]. Incorporation of these nucleotides into DNA or RNA, still allows the formation of a double-helix structure for both molecules [91]. The use of DNA containing codons based on an expanded genetic alphabet opens the possibility of establishing an expanded translation system that also allows the incorporation of ncAAs into proteins.

Another strategy for alternative base-pairing relies on the development of size-expanded nucleotides. By the addition of a benzene ring, both purines and pyrimidines can be expanded by 2.4 Å, and when paired to dNTPs they still enable the formation of a double helix. The resulting molecule, called xDNA, is wider than the natural double helix and has a higher stability than a DNA of analogous sequence, mostly due to the enhanced stacking of the enlarged nucleobases [92]. xDNA was evaluated for its ability to function like a genetic system *in vivo* [93]. Plasmids encoding green fluorescent protein were constructed to contain up to eight xDNA bases and were transformed into *E. coli*. All four xDNA bases were found to pair correctly in the replicated plasmid DNA, and also permitted the expression of the fluorescent protein, turning colonies green. Based on the evidence, xDNA could function as an artificial genetic system, however the enzymatic machinery able to efficiently polymerize these nucleotides is yet to be developed.

When it comes to artificial nucleobases, complementary hydrogen bonding is not a condition for the efficient and selective replication of DNA [94]. Metal-dependent pairing as well as hydrophobic and packing forces (including ring-stacking) have been explored [95,96]. Two promising candidates for an alternative base-pairing system based on hydrophobic nucleotides have been described (d5SICS-dNaM and d5SICS-dMMO2). Both artificial pairs are efficiently transcribed in both directions [97] and can be amplified by PCR [98]. A crystal structure of a KlenTaq DNA polymerase bound to a DNA containing the pair dNaM-d5SICS showed that the structure adopted by the modified nucleobases is co-planar with nearly optimal edge-to-edge, similar to what is observed in DNA [99].

In order to test how feasible it would be to expand an organism's genetic alphabet *in vivo*, Malyshev and colleagues designed a system for *E. coli* based on the incorporation and replication of the dNaM-d5SICS nucleotide pair. *E. coli* was transformed with two plasmids, one carrying a gene for the expression of a heterologous nucleoside triphosphate transporter for the active uptake of dNaM and d5SICS, and a second plasmid carrying a sequence with the six-letter code (dA, dT, dC, dG, dNaM and d5SICS) to work as a template for replication. In what represented a major breakthrough in the field, both modified nucleotides were taken up from the culture media, efficiently incorporated into the newly synthesized copies of plasmid, and remained untouched by the *E. coli* DNA repair mechanisms for about 24 generations (17h) [100]. This work can now be expanded to integrate the modified base-pairs into other cellular processes such as translation–as demonstrated by Fukunaga and colleagues for a different set of unnatural nucleobases [101].

However, whether XNAs will be able to sustain life is still an open question. In this regard and in contrast with most of the studies performed so far, Marlière and colleagues carried out a top-down experiment designed to perform the DNA transliteration of the entire *E. coli* genome. A strain deficient in the synthesis of thymine nucleotides was cultured in the presence of 5-chlorouracil as an alternative and artificial nucleobase precursor. After 1000 generations and more than 1500 mutations, an adapted strain that grew only with 5-chlorouracil was recovered. Its genome contained 90% of the 5-chlorodeoxyuridine and 10% of the thymidine, which was later reduced to 1.5% (the limit of detection) by disrupting a secondary pathway for the synthesis of thymidine based on S-adenosylmethionine [102]. To date, this is the sole report of an organism whose genome carries a modified nucleotide in place of a natural one, suggesting that XNA-based life is possible.

## Minimal genomes

There is a different subfield in synthetic biology that works at the whole genome level. Current approaches on genome-driven cell engineering aim to develop functioning minimal genomes that only contain the genes that are necessary to sustain life. The development of minimal genomes entails the screening of natural genomes to find candidates that could be then devoid of non-essential genes. The aim is to create a simple standardized host cell that would allow the implantation of synthetic biology devices. Although chromosomes in here are still envisaged as DNA based, a minimized genome should limit the potential of the host to adapt to a different environment and growth conditions. Thus, the likelihood of perpetuating and evolving if the microbe is released into the environment would be reduced [103]. In this regard, an engineering strain of *Mycoplasma mycoides* bacterium with the smallest genome and the fewest genes of any freely living organism was reported earlier this year [104]. This newly engineered organism was named Syn 3.0 and it has a genome that was reduced to 473 genes required to survive and reproduce. Syn 3.0 colonies are morphologically similar to those of the original cell although smaller, probably due to its slower growth rate (Syn 3.0 doubling time is three hours in comparison with one hour for the original cell).

The arrangement of a minimal DNA genome carrying only ‘housekeeping’ genes combined with an XNA-based episome engineered to provide traits of interest would allow the development of enhanced safeguard systems.

## Mechanistic containment

A final approach to biological containment is the development of genetic elements that require mechanisms orthogonal to the host to be viable. In a basic sense, mechanistic orthogonality pertains to the inability of some natural enzymes (e.g. polymerases) or even higher complexes (e.g. ribosomes) to access foreign–natural or synthetic–substrates.

Examples of natural genetic elements that are mechanistically orthogonal to the cellular genetics include many phage and viral chromosomes, which are replicated only by means of their self-encoded polymerases [105]. Host-cell polymerases are subjected to mechanistic inhibition, as they are unable to initiate replication of the foreign genetic unit. This can be adapted for containment by establishing systems where the replicase is provided *in trans*. Though its efficiency would be presumably lower than other genetic or semantic containment, mechanistic orthogonality could be an enabling tool for both of these.

All three of the fundamental processes of the central dogma–DNA replication, transcription and translation–are most highly regulated at the stage of initiation [106–110]. Initiation is the major targeting step for signalling pathways triggered by stress factors, such as heat shock and pathogens. This is most probably related to the high energetic cost these processes have (especially translation). The burden of placing regulatory checkpoints too far downstream would be not viable due to the associated metabolic cost.

Consequently, it is possible to engineer systems that rely on orthogonal modes of initiation of the replication or expression processes (e.g. T7 transcription, see below). A system like this would allow an engineered enzyme to catalyse an early-stage step on a foreign target that would otherwise be inaccessible to the host. If the survival of the host is then linked to its ability to maintain a foreign episome and transcribe a foreign open reading frame, or translate a foreign mRNA, it would be possible to develop cells with a second pool of genetic material and/or ribosomes.

However, few such systems exist, and many retain a high level of cross-talk with native substrates. For instance, as described in previous sections, several polymerases have been engineered to incorporate xNTPs at the elongation stage. For *in vivo* applications, however, the currently available enzymes are not suitable, as they tend to retain activity on their natural substrates, and in many cases this outcompetes the XNA synthesis activity (C. Cozens and V.B. Pinheiro, personal communication). Thus, incorporating orthogonality into the initiation step would be essential to separate the activity of an engineered enzyme from its natural substrate, preventing cross-talk with host substrates. Such isolation is important in synthetic biology because it allows the isolated system to be engineered without a significant impact on the rest of the cell. In the case of an orthogonal episome, this would imply being able to engineer enzymes, cofactors and open reading frames encoded in the episome free from the burden of having to sustain the host. As a result, such isolated systems could be more readily engineered (i.e. with fewer evolutionary constraints) than systems that are required for host survival.

## Orthogonal replication as a containment strategy

The simplest system of this kind is therefore one in which a foreign episome is replicated by an orthogonal mechanism, as described previously in the genetic containment section.

The most commonly used genetic elements in *E. coli* molecular biology are plasmids. However, for the purposes of containment, they do not allow orthogonality as they are replicated by host factors [111].

In recent years, two replication systems for linear chromosomes that are independent of the host enzymatic machinery have been characterized and assembled. These are the replisome of the bacteriophage phi29 that infects *Bacillus subtilis* [112] and the selfish replicating system of the linear plasmids pGKL1 and pGKL2, which infect certain strains of the yeast *Kluyveromyces lactis* [113]. Both systems utilize a polymerase that exclusively replicates linear genetic elements with the aid of certain accessory proteins such as priming proteins (also known as terminal proteins or TP), and single-strand and double-strand DNA binding proteins [114,115]. The phi29 DNA polymerase does not initiate replication from an RNA primer but from the serine hydroxyl-group of the TP protein [116]. In infected cells, the polymerase exists as a dimer with TP [117,118] and only dissociates after initiation, when the TP remains covalently attached to the end of the phage genome. Although *in vitro* the polymerase can extend a primer on a single-stranded template, its efficiency is reduced in the absence of its accessory proteins (single-strand and double-strand DNA binding proteins and TP) [112].

A phi29 replisome would be powerful *in vivo* since in the presence of TP, the polymerase can be exclusively targeted to the replication of linear genetic elements that are capped by TP. Though the system is not yet adapted for the *in vivo* replication of heterologous elements, it is a potential platform for the development of orthogonal genetic elements. An alternative phi29 *in vitro* episome has been developed based on rolling circle amplification, but it cannot in its current state be used for *in vivo* applications, since DNA replication does not regenerate the discrete genomes like the input genome (input: single-stranded circular; output: single-stranded linear), and is not orthogonal as it is able to use primers generated by RNA polymerases [119].

In the case of the *K. lactis* pGKL1/2 system, which is expected to replicate its genome in a similar way to phi29 [113], there is an additional physical separation in the infected cells between the plasmid genome in the cytosol and the host genome in the nucleus. The encapsulation of cellular processes within membranous compartments such as the nucleus allows the spatial separation–or compartmentalization–of the host genome from the secondary episome. This or similar methods could therefore also contribute to the orthogonality of engineered episomes based on alternative genetics, where it may be essential to avoid cross-talk, especially of the engineered polymerases (see genetic containment; point 6). Alternative compartmentalization schemes include proteinaceous microcompartments, in which a number of metabolic pathways have been engineered [23].

## Orthogonal translation as a containment strategy

As found in transcription, where promoters and polymerases can be modified to be orthogonal to the host systems (discussed above), regulatory elements in translation and the cellular translation machinery itself can be engineered to isolate a circuit from wider biology.

Translation initiation in prokaryotes is regulated by the binding of the 30S ribosomal subunits (with associated initiation factors) to ribosomal binding sites (RBSs, also known as Shine-Dalgarno sequences), located within the substrate mRNA upstream of initiation codons [120,121]. RBS sequences are reverse complementary to the 3’-end of the 16S ribosomal RNA of the 30S subunits (the anti-SD) and rely on base-pairing for the initial interaction. Rackham and Chin exploited that requirement to diversify anti-SD sequences in the 30S of ribosomes, isolating variants (O-ribosomes) with modified RBS requirements that could not initiate translation from the canonical RBS (i.e. were orthogonal to the biological system) [122].

Freed from their biological roles, these ribosomes have been exploited to increase yields obtained from ncAA-containing protein synthesis [123] as well as to further engineer the ribosome itself [48,124–126]–with a potential impact on genetic code engineering (see below).

Being more complex than its bacterial counterpart, the eukaryotic translation system may provide additional potential processes that can be targeted for containment by creating a platform that can coexist but not interact with the natural cellular processes. Although no orthogonal ribosome has been isolated in eukaryotes, there is mounting evidence that there are distinct populations within cells [127,128] and that they can be engineered [129].

An alternative approach is to engineer the reading window required by the ribosome. Although the genetic code is exclusively based on triplet codons, a number of tRNA variants have been reported that induce a frameshift when incorporated in translation (see Figure 2) [130–133]. These variants expand the tRNA contact with the mRNA leading to a quadruplet codon, which can be incorporated by natural systems in some sequence contexts and may provide the host with a marginal evolutionary advantage by increasing the coding potential of some of its genes [134–136].

Quadruplet codons have been exploited for the incorporation of ncAAs in proteins [124,137] and their incorporation can be further improved by engineering the ribosome itself [48,123]. Quadruplet codons not only increase the available genetic code (256 rather than 64 possible variations) but could also have a role in containment where this mechanistic containment would also generate a semantic containment with the synthesis of functional proteins dependent on a codon space not naturally available.

## Conclusions

The synthetic biology industry is one of the fastest growing industries, projected to be worth 5 billion dollars by 2018 [138], with chemicals, pharmaceuticals, energy and agriculture, as some major application markets. The increased reliance on biology as an industrial tool, together with the potential to synthesize organisms from scratch, decreases the probability that we understand and can predict every possible interaction between engineered hosts and the environment. For the sustained growth of the bioeconomy and for our own safety, it is important that biocontainment evolves from tackling the known risks to pre-empting unknown risks and routes of escape. In order to develop containment mechanisms built-in within the GEM itself, synthetic biology has deployed a collection of tools that are driving the design of a new generation of biocontainment strategies. These mechanisms intend to be not only more effective than previous strategies but also potentially intercompatible. There is agreement in the synthetic biology community that multiple and redundant systems of biocontainment are necessary to prevent escapes, with containment not resting on any single component that can be bypassed by inactivation (i.e. by mutations) [139]. The development of multi-layered control systems that can coexist within a single engineered microorganism should hopefully reduce the probability of an escape to negligible levels. We are still some way from robust orthogonal systems that can be used for practical applications; not only does the enzymatic machinery necessary to deal with these artificial systems still need to be designed, but true orthogonality via the development of “no cross-talk” firewalls also has to be guaranteed.

## Summary

- Biology is not limited by what is natural but by what is possible. Life emerged from the chemistry permissible by available substrates. Natural evolution builds on that, with rare instances of innovation and multiple instances of optimization. Consequently, it is possible to engineer a biological system to access unnatural substrates or generate unnatural products, which may be of value to humanity (e.g. novel materials, anti-cancer or antimicrobial compounds or biofuels).
- Genetically engineered microorganisms (GEMs) present two potential environmental hazards, ecological or informational, depending on whether the organism can become established in an ecological niche or its genetic information can be transferred to a natural host.
- Risk can be accurately measured only if all possible failure modes and their probabilities of occurrence are known.
- Biological containment is a long-standing concern for scientists. A number of containment strategies have been developed and are widely used by the academic and industrial communities. These are centred primarily around physical barriers, use of microorganisms with decreased fitness and genetic circuits that limit the escape of genetic information.
- All biological processes can be altered to establish systems that do not exchange information with nature, be it genetic information storage, its maintenance, protein translation or even the cell's metabolism. These orthogonal systems can be the basis of a new generation of biological containment strategies where both organism and information are isolated from the environment and unable to interact with biology.

## Abbreviations

aaRS: aminoacyl-tRNA synthetase
BFA: L-4,4′-biphenylalanine
CeNA: cyclohexene nucleic acid
CFU: colony forming unit
DNA: deoxyribonucleic acid
dP: 2-aminoimidazo[1,2-a]-1,3,5-triazin-4(8H)-one
dZ: 6-amino-5-nitro-2(1H)-pyridone
GEMs: genetically engineered microorganisms
GMOs: genetically modified organisms
GNA: glycerol nucleic acid
HNA: 1,5-anhydrohexitol nucleic acid
LNA: locked nucleic acid
mRNA: messenger RNA
ncAA: non-canonical amino acids
O.D.: optical density
pAF: p-aminophenylalanine
PCR: polymerase chain reaction
RBS: ribosomal binding site
RF: release factor
RNA: ribonucleic acid
SD: Shine-Dalgarno
TNA: threose nucleic acid
TP: terminal protein
Tpa: L-beta-(thieno[3,2-b]pyrrolyl)alanine
tRNA: transfer ribonucleic acid
XNA: xenobiotic nucleic acid
xNTP: xenobiotic nucleoside triphosphate
